# The impact mechanism of China’s green finance on the transformation and innovation of high-energy-consumption enterprises

**DOI:** 10.1371/journal.pone.0293022

**Published:** 2023-10-16

**Authors:** Weimin Xiang, Yeqiang Lan, Qiao Qi, Lei Gan

**Affiliations:** 1 School of Management Science and Engineering, Chongqing Technology and Business University, Chongqing, China; 2 School of Management Science and Real Estate, Chongqing University, Chongqing, China; East China Normal University, CHINA

## Abstract

The development of green finance and the promotion of green transformation and upgrading of high energy-consuming enterprises are of great significance for China to achieve the "double carbon" goal. This paper employs a dual fixed-effects model to examine the profound ramifications and intrinsic mechanisms of green finance development on the transformative innovation of high-energy-consumption enterprises, using a sample of 462 publicly traded high-energy-consuming corporations from the period spanning 2016 to 2020. The results show that the development of green finance promotes the transformation and innovation of energy-intensive enterprises and that market-incentivized environmental regulation plays a partially mediating role; the results of heterogeneity analysis show that green finance promotes the transformation and innovation of high energy-consuming enterprises with significant differences in different low-carbon pilot regions, company ownership, and enterprise size; the mechanism analysis shows that the development of green finance can increase government subsidies and alleviate financing constraints to promote the transformation and innovation of high energy-consuming enterprises; it is also found that the development of green finance can significantly improve the financial performance of enterprises. The research findings of this paper hold significant implications for promoting the sustainable development of green finance and high-energy consumption enterprises in China. They provide valuable insights and references for facilitating the green transformation and innovation of high-energy-consuming enterprises in China as well as other developing countries.

## 1. Introduction

China has had remarkable economic growth during the decades of reform and opening up, and the degree of innovation has also risen significantly. However, crude economic growth has also brought a series of environmental problems, and the issue of the green transformation of enterprises is highly valued. As global temperatures rise and greenhouse gases are emitted in large quantities, climate issues are evolving into one of the greatest challenges to human development. In 2020, General Secretary Xi stated at the United Nations General Assembly that China should aim to achieve carbon peaking by 2030 and carbon neutrality by 2060. The "double carbon" objective will further encourage the green transformation of China’s heavily polluting and energy-intensive industries as well as the low-carbon and sustainable growth of its economy (According to the National Bureau of Statistics of China, the National Economic Classification of Industries, and the National Economic and Social Development Statistics Bulletin, this paper identifies six categories of high energy-consuming industries, including petroleum, coal, and other fuel processing industries, chemical raw materials and chemical products manufacturing, non-metallic mineral products industry, ferrous metal smelting, and rolling processing industry, non-ferrous metal smelting and rolling processing industry, electricity, heat production, and supply industry.). In the first half of 2023, the growth rate of the value added in China’s high-energy-consuming sector notably decelerated. However, the investment structure still requires further enhancement, and the ongoing transformation efforts in clean technology remain imperative. In such a context, China needs to further increase financial support and improve the system related to green finance. This requires green finance to adapt to the new environment and development situation as soon as possible, guiding and urging enterprises to actively assume social responsibility while promoting their green transformation and innovation, and fostering the "double carbon" goal’s early realization. As a result, green finance’s swift development has emerged as a crucial strategy to boost innovation and the development of corporate green transformation.

Green finance refers to the financial services provided for projects related to green, low-carbon, and energy-efficient sectors, aiming to support environmental improvement and efficient resource utilization. In comparison to traditional finance, green finance places a greater emphasis on the benefits of human society’s survival environment. Moreover, it guides economic entities to prioritize the maintenance of natural ecological balance through their own activities. As an important force in mitigating environmental pollution, green finance is crucial for improving environmental conservation and lowering environmental pollution [1, 2]. Existing literature on green finance predominantly focuses on studying the impacts it generates on the ecological environment, economic development, energy consumption, and other related aspects [3, 4]. As the scale of green finance development expands, the scale effect, structural effect, and environmental regulation significantly improve the regional carbon emission reduction effect and the ecological environment [5]. As a key element of green finance, green credit has greatly reduced China’s rate of carbon emissions, and its effect differs significantly in the east, west, and central parts of China [6]. Meanwhile, the differentiated green credit policy has guided enterprises to strengthen their management efficiency and promote low-carbon technology innovation in non-green enterprises, thus realizing the dual optimization of industry and technology [7]. The above illustrates that green finance is actively pushing the decarbonization of China’s economy. In addition, green finance guides enterprises from high-pollution and high-energy-consuming industries to low-carbon cycle development and generates corresponding scale, structure, technology, and other effects to bring capital for the green transformation of enterprises [8–10]. Among them, the actual operations of high polluters are harmed by green credits due to financial constraints, which to some extent inhibit their emissions behavior but do not actively seek transformation and upgrading through technological innovation [11].

The green transformation and innovation of enterprises are based on the specific situation of the existing economic and social development and the degree of tolerance of resources and the environment, guided by the concept of green development, and through changes in the mode of operation and industrial structure of enterprises to make their development independent of inputs from resources and the environment, thus realizing transformation and upgrading. This kind of green transformation and innovation can not only decrease environmental contamination and boost business performance, but more importantly, they can produce diversified products and improve their core competitiveness, thus attaining the "win-win" of economic benefits and environmental preservation [12]. Specifically, companies receive a good environmental reputation while reducing their own material and energy use costs, capital costs, and labor costs, thus contributing significantly to their green profitability [13]. Against the backdrop of sustainable development, the quality of innovation in heavily polluting companies has been affected, and green bonds have significantly contributed to the quality of innovation and the level of transformational innovation [14]. Other scholars have found that green finance significantly reduces firms’ financing costs and credit constraints, thus promoting the sustained progress of green firms [15]. Meanwhile, the implementation of green lending policies may limit investments in energy-intensive industries or industries with high pollution and energy consumption, thus contributing to the green transformation of enterprises [16]. In addition, green finance can effectively tackle issues with business development and improve economic efficiency by facilitating the financing of capital [17, 18]. It can be observed that the effective promotion of green transformation and innovation in enterprises is an important criterion for measuring the magnitude of the role of green finance. However, there is limited research specifically targeting high-energy-consumption enterprises. These enterprises have a significant proportion of energy consumption and emit substantial amounts of pollutants. Finding ways to both curb their pollution emissions and incentivize increased research and development investment is a crucial pathway for China to achieve its "dual-carbon" goals and promote sustainable socio-economic development. Therefore, this paper primarily focuses on the research and exploration of the impact and mechanisms of green finance on high-energy-consumption enterprises. Additionally, it further investigates the influence of green finance on the economic and environmental benefits of these enterprises.

This article contributes as follows: Firstly, existing studies on the impact of green financial resources on the transformation and innovation of high-energy-consuming enterprises are uncommon and often overlook the effects and underlying mechanisms of green finance on the transformation and innovation outcomes of these enterprises. This essay delves into the impact of green finance on the transformation and innovation of high energy-consuming companies using microdata from these companies and also examines its mechanisms of effects and indirect effects, providing valuable insights for subsequent analyses. Secondly, this paper focuses on the economic and environmental advantages of high energy-consuming enterprises, and the intrinsic mechanism of green financial support intensity for high energy-consuming enterprises is systematically analyzed. Existing research often overlooks the impact of enterprises on the environment and their own economic development [19]. This study further enriches the existing body of knowledge by addressing these aspects and providing additional insights. Thirdly, in the analysis of heterogeneity, this study adequately explores the heterogeneous impacts on high-energy-consumption enterprises based on different low-carbon pilot regions, ownership structures, and firm sizes. This provides robust support for the long-term development of high-energy-consumption enterprises in the green context. In addition, this study investigates the moderating effect of financing constraints and government subsidies on the influence of green finance on the transformation and innovation of high-energy consumption enterprises. This is because the flow of green finance resources is closely related to the efficiency of financial resource allocation, and government intervention that is misguided can potentially lower the efficiency of resource allocation. Once efficiency is reduced, it can exacerbate financing constraints for enterprises, thereby hindering their green transformation and innovation [20].

The remaining structure of this paper is organized as follows: Section 2 provides a comprehensive review of the relevant literature, includes theoretical analysis, and outlines the research hypotheses. Section 3 provides an explanation of the model, variable selection, data sources, and the econometric model. Section 4 presents the empirical findings of the study. Section 5 discusses the economic and environmental advantages resulting from the development of green finance and compares them with findings from other relevant studies. The conclusions and policy recommendations are presented in Section 6.

## 2. Literature review and research hypothesis

### 2.1 Literature review

Currently, there is no globally unified and explicit definition for green finance, and further improvements and expansions are still needed in the development of the green finance system [21]. In China, where financing restrictions are relatively strict, green finance has become a financial system that combines environmental protection with economic interests [1], playing a significant role in environmental conservation and the sustainable development of the green economy. For example, Saeed Meo and Karim [4] discovered that there is a positive relationship between green finance and carbon dioxide mitigation. They concluded that green finance proves to be an effective financial strategy for reducing carbon emissions. Furthermore, green finance can effectively mobilize a company’s financial resources to achieve carbon reduction [22]. Similar to traditional finance, the development of green finance contributes to economic growth [23] and enhances green total factor productivity [24]. Additionally, green finance is a significant force in promoting industrial structural optimization by optimizing resource allocation and directing funds towards green and ecological development [25]. Several studies also provide empirical evidence that green finance facilitates corporate transformation. For instance, Li et al. [26] found that green finance changes the traditional production methods of enterprises, enabling them to undergo a green transformation and engage in more environmentally friendly production. According to the research conducted by Hsu et al. [27], green finance has the capacity to efficiently regulate the allocation of financial market resources and share research and development risks with enterprises. As a result, green finance plays a crucial role in promoting green innovation.

The ability of enterprises to achieve green transformation and innovation is key to China’s further sustainable development. Green finance influences the opportunity cost of pollution for enterprises by adjusting the allocation of capital, compelling them to increase green investments and reduce pollution-related investments, thus promoting their green transformation. Research has found that when companies invest in environmental technology research and development, green finance can help them obtain green financing [28]. Additionally, green finance provides financial support for enterprises’ green transformation and innovation. When companies spill the beans on their environmental information and get their hands dirty with green innovation, their financing situation can see some real improvement [29]. Zhang et al. [30] conducted a study using the Green Credit Policy (GPC) as a real-life test and discovered that the GPC actually beefed up the access to financing and credit support for those heavy-polluting and energy-guzzling companies. According to Jia [31], through a thorough examination of companies’ financial limitations, it was revealed that the implementation of green credit leads to a surge in innovation efforts among enterprises by easing their external financing constraints. Liu and Xiong [32] discovered that the reform of green finance significantly promotes corporate innovation. Lowering their debt costs and increasing innovation investment further strengthens this effect, which is particularly significant for local state-owned enterprises and companies with lower online attention. Moreover, research has shown that when companies have guaranteed profitability and access to external funds, green finance enhances their level of green innovation [33]. As the green finance system becomes increasingly refined, the reduced costs for financial institutions promote enterprises’ green transformation and innovation [34].

From the discussions above, most of the existing literature focuses on the direct effects of green finance. This includes its impacts on the environment, economic development, and industrial structural optimization. Some studies also explore the effects of green finance on high-polluting enterprises. For instance, green finance guides financial resources to flow from high-polluting enterprises to green and environmentally friendly enterprises, promoting the development of clean green technologies and facilitating green transformation [35]. It is worth considering that government environmental regulations are an important factor that should not be overlooked when studying the transformation and innovation processes of high-energy-consumption enterprises. Considering the relatively low proportion of green investments in China’s current fiscal expenditure, existing research may overlook the practical situation of green transformation and innovation in Chinese enterprises. Moreover, there’s a notable gap in comprehensive research regarding the impacts and underlying mechanisms of green finance on the transformation and innovation outcomes of high-energy-consumption enterprises. There’s a need for further exploration to comprehend whether green finance can indeed effectively drive the transformation and innovation of high-energy consumption enterprises, as well as to identify the specific pathways through which this can be achieved.

### 2.2 Hypothesis development

Green credit, as the main part of China’s green finance, enables banks and other financial agencies to adjust the flow of financial resources according to the corresponding policies. In addition, green credit further increases funding for environmental protection and green projects while also imposing corresponding credit constraints on projects with high emissions, high energy consumption, and high pollution [36, 37]. On the one hand, green credit guides enterprises to develop low-carbon and green projects, and the corresponding production resources are gradually tilted towards environment-friendly enterprises, reducing their financing costs and optimizing resource allocation, thus improving the corresponding conditions for companies to transform and innovate accordingly [38]. On the other hand, the recognition of green finance by external investors and the higher attention of the market under the strong advocacy of the concept of sustainable development have brought some convenience to external financing for non-green enterprises [39]. Investors are also choosing to focus more on projects that achieve both sustainability and performance, thus prompting non-green companies to make green transformations and innovations for long-term growth. Therefore, hypothesis H1 is proposed.

H1: Green finance remarkable promotes the transformation and innovation of high-energy-consuming enterprises.

The vigorous development of green finance favors green environmental projects that promote sustainable social development, but the imbalance in development between regions in China has caused green finance to remain in an immature stage. Solving this challenge requires the government to involve the corresponding environmental regulations in the construction of the green finance system. At present, China’s environmental regulations are relatively well developed, and the standard of sewage charges levied on enterprises in this regard has approximated the cost of pollution treatment for the enterprises themselves [40]. Neoclassical theory suggests that increased environmental regulation exacerbates firms’ production input costs and reduces their profit margins. Firms are driven by profit maximization and have to crowd out their own R&D investment, thus hindering the development of innovation capabilities [41]. With the gradual increase in environmental awareness in society as a whole and among enterprises, more companies are willing to make green transformations and innovations rather than pay for their emissions. Under the environmental regulations of "command and control" and "market incentives", high pollution and high energy consumption require high emission fees, which take away part of the funds for R&D and operation. As a supplement and regulation in the process of green finance influencing the transformation and innovation of high energy-consuming enterprises, environmental regulations can effectively combine with the local actual situation to restrain and regulate the green financial services provided by financial institutions [42]. Therefore, hypothesis H2 is proposed.

H2: There is an indirect effect of environmental regulation in the process of green finance on the transformation and innovation of high-energy-consuming enterprises.

The innovation of enterprises is often characterized by high investment and high risk, suggesting that the green transformation and innovation of enterprises need sufficient financial support and also need to solve problems such as serious financing constraints. The essence of green finance is the configuration of resources for environmental information, and its development is inseparable from the support of government policies and a certain amount of financial subsidies. Past studies have found that green credit is an essential way for green finance to provide credit support to green enterprises [43]. Not only does it improve the debt, financing structure, and long-term borrowing of green companies, but it also significantly eases their financing constraints [44]. Besides, government subsidies to enterprises can also serve to alleviate their financing constraints and thus provide incentives for green transformation and innovation activities. Therefore, hypothesis H3 is proposed.

H3: Increasing government subsidies and easing financing constraints can significantly promote the effect of green finance on the transformation and innovation impact of high-energy-consuming enterprises.

The promotion of green finance can help accelerate the pace of China’s sustainable economic development and promote the harmonious development of the ecological environment. Firstly, the vigorous implementation of green credit and green bonds can improve the long-term value and business performance of enterprises [45]. Secondly, the corresponding green transformation of enterprises can not only bring a good reputation for themselves but also help them save on materials, labor, and other costs, thus realizing certain green benefits [46].

However, due to the difficulty of green innovation projects that are entirely focused on improving the environment and reducing pollution, Chinese companies are currently struggling to meet the requirements of improving their own environmental performance. There is still a significant reliance on government subsidies by many enterprises, which may hinder the development of green and clean technologies. Additionally, developed countries generally exhibit better environmental performance by their enterprises, which can be attributed to factors such as the earlier implementation of green finance, higher economic levels, and more advanced technologies [47]. Therefore, the effect of green transformation innovation by energy-intensive enterprises to improve their environmental performance under the current situation may not be obvious yet. Therefore, hypothesis H4 is proposed.

H4: The transformation and innovation of energy-intensive enterprises can significantly improve their financial performance, but the impact on environmental performance in the short term is not significant.

**Fig 1:**
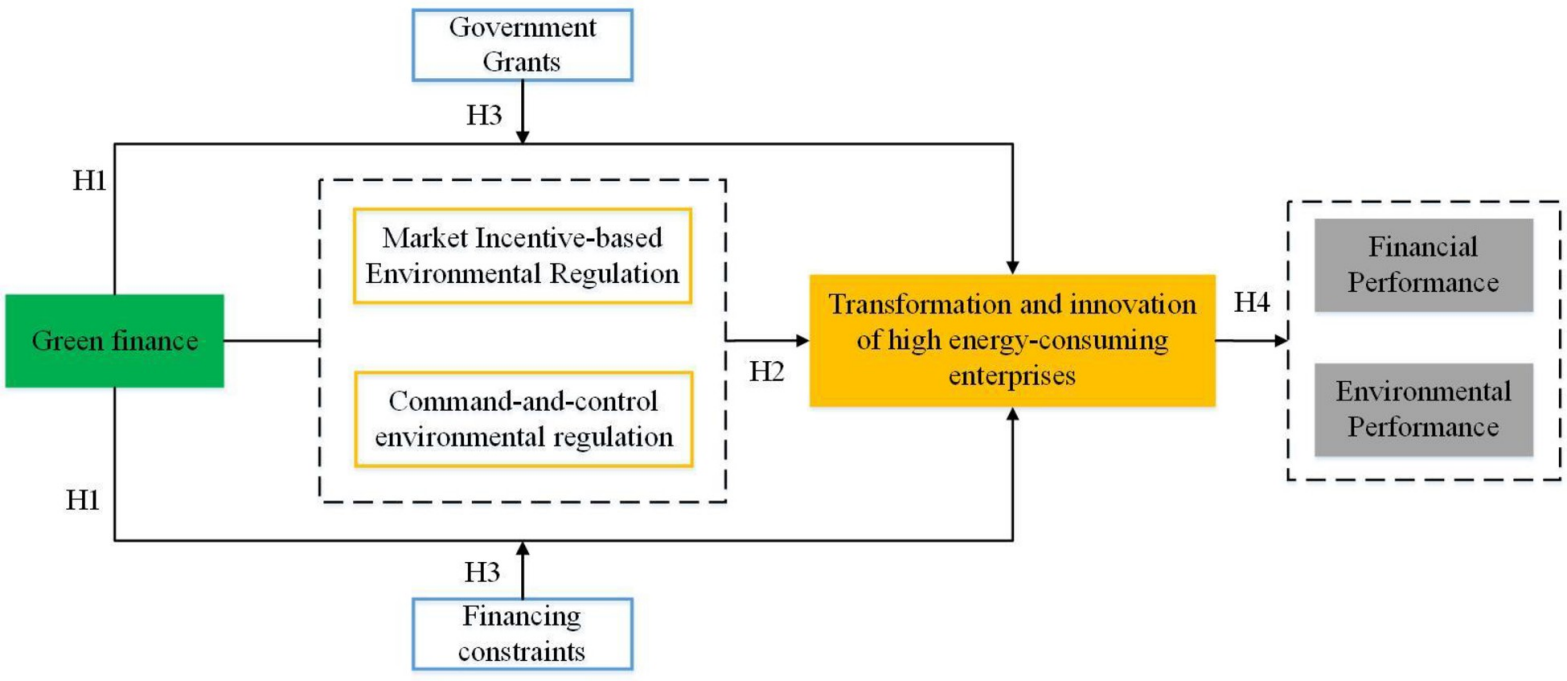
Study framework.

Based on the above, the study framework of this article is shown in Fig 1.

## 3. Methodology

### 3.1 Research design

The two-way fixed-effects model is widely used in social science research and aims to analyze the interaction effects between two or more different levels. In order to comprehensively and accurately investigate the impact of green finance on the transformation and innovation of high-energy consumption enterprises while considering the unobserved heterogeneity in the sample that may vary across individuals and over time and its impact on the regression results, this study employs the two-way fixed-effects model as the foundational econometric model. The econometric model is constructed as follows:

$${TL}_{it}=\alpha_{0}+\alpha_{1}{GreenFin}_{it}+\alpha_{2}{Control}_{it}+\varphi_{i}+\gamma_{t}+\varepsilon_{it}$$ (1)

*TL*_*it*_ represents the level of transformational innovation of high energy-consuming firm *i* in year *t*; *GreenFin*_*it*_ denotes the green financial support obtained by firm *i* in year *t*; *Control*_*it*_ denotes the control variable; *φ*_*i*_, *γ*_*t*_ denote individual, year fixed effects; *ε*_*it*_ denotes the stochastic interference term.

To further explore the indirect effects of environmental regulation, a three-step approach with stepwise test coefficients is used [48]. Construct the following model:

$$M_{it}=\alpha +\rho_{1}{GreenFin}_{it}+\rho_{2}{Control}_{it}+\varphi_{i}+\gamma_{t}+\varepsilon_{it}$$ (2)

$${TL}_{it}=\alpha +\eta_{1}M_{it}+\beta_{1}^{\prime }{GreenFin}_{it}+\eta_{3}{Control}_{it}+\varphi_{i}+\gamma_{t}+\varepsilon_{it}$$ (3)

*M*_*it*_ is the mediating variable in the above model; model (2) mainly verifies the effect of green finance on the mediating variable; model (3) explores the indirect effect of the mediating variable; and the rest of the variables are in agreement with the preceding.

To test the influence mechanism of government subsidies and financing constraints on the transformation and innovation of high energy-consuming enterprises, this paper adds two variables, government subsidies, and financing constraints, to the regression, and also brings government subsidies (GS) and its interaction term with green financial support strength, and the interaction term between financing constraints and green financial support strength into the model together for regression, and reference to existing literature [49], the financing constraint of high energy-consuming enterprises is measured by the FC index, and a larger value indicates a greater degree of financing constraint, and the model is as follows:

$${TL}_{it}=\alpha +\mu_{1}{GreenFin}_{it}+\mu_{2}{FC}_{it}+\mu_{3}{FC}_{it}\times {GreenFin}_{it}+\mu_{4}{Control}_{it}+\varphi_{i}+\gamma_{t}+\varepsilon_{it}$$ (4)

$${TL}_{it}=\alpha +\omega_{1}{GreenFin}_{it}+\omega_{2}{GS}_{it}+\omega_{3}{GS}_{it}\times {GreenFin}_{it}+\omega_{4}{Control}_{it}+\varphi_{i}+\gamma_{t}+\varepsilon_{it}$$ (5)

Where *GS*_*it*_ represents government subsidies, *FC*_*it*_ represents financing constraints, and the rest of the variables are in agreement with the preceding.

### 3.2 Data and sample selection

This study selects listed companies in the high-energy-consuming industry within the scope of investigation and examines the level of green finance support provided by various provinces (cities, autonomous regions) during the period from 2016 to 2020. The following treatments were also carried out: Initially, deletion of ST-category enterprises Additionally, to avoid significant biases in the results, enterprises with a high degree of missing data were excluded. Furthermore, elimination of enterprises with less than 3 consecutive years of core data and double-sided tailing for continuous variables In addition, the relevant financial data are mainly from the Chinese Economic and Financial Research Database (CSMAR) database (https://cn.gtadata.com/); the data on the green financial support strength of each province are from the China Industrial Statistical Yearbook, the China Economic Census Yearbook, etc.; the data for environmental regulation are from the China Environmental Statistical Yearbook (http://www.stats.gov.cn/), the China Tax Yearbook (http://www.chinatax.gov.cn/), and the Wind database (https://www.wind.com.cn/); and finally, 2310 samples were obtained.

#### Dependent variable

The firm’s level of transformational innovation (*TL*). In the current study, most scholars measure the level of transformational innovation based on firms’ innovation inputs and outputs. The ratio of R&D expenditure to operating income is used in terms of innovation investment, which to a certain extent reflects the degree of transformation and innovation in the company. The continuity feature of R&D expenditure facilitates the allocation of innovation resources and directly affects the innovation capacity of enterprises [50]. In terms of innovation output, the number of green patents authorized can best reflect the effectiveness of transformation and innovation in enterprises. This paper argues that green finance guides high-energy-consuming enterprises to transform and innovate by allocating resources, and the level of transformation and innovation is measured by the ratio of R&D expenditure to the operating income of enterprises. The greater the R&D investment, the more it reflects the efforts made by the company at the level of R&D and innovation, as well as the transformation and innovation capabilities of high-energy-consuming enterprises.

#### Independent variable

Green financial support (*GreenFin*). Green finance has developed rapidly in a dual-carbon context, with high-energy-consuming companies investing heavily and consistently in participating in green development innovations and relying heavily on external funding. Under the concept of green sustainability, more financial resources are being directed to the green sector, which makes it more challenging for non-green companies to obtain external financing. Meanwhile, the concept of green development has accelerated the green transformation and innovation of high-energy-consuming and high-polluting enterprises, guiding high-energy-consuming enterprises to develop more energy-saving and environmentally friendly projects to reduce environmental pollution. In this study, the proportion of interest expenses of non-high-energy-consuming enterprises within total industrial interest expenses is used as an indicator of green finance [51].

#### Intermediary variables

Command-based environmental regulation (*ERc*), market incentive-based environmental regulation (*ERm*). Based on the different subjects of environmental regulation, most of the existing studies classify them into three categories: first, command-and-control environmental regulation, which refers to the government’s enforcement of laws and other coercive measures to control activities that are harmful to environmental development and will be punished if they do not meet the corresponding standards set by the government; the second is market-incentivized environmental regulation, in which the government uses the "polluter pays" principle to get companies to control their environmental pollution; the third is mass participation environmental regulation, which reflects the environmental awareness of the citizens and curbs their polluting behavior by putting pressure on the government and polluters. Considering that the subject of this article is the firm, it needs to directly deal with the environmental regulations related to enterprises. Therefore, command-based and market-incentive-based environmental regulations are adopted as mediating variables, and the former is measured by the emissions of "three wastes" measured by the entropy method, and the latter is measured by the logarithm of sewage charges and environmental taxes. The higher the values of *ERc*, the lower the strength of command-based environmental regulation, and the higher the values of *ERm*, the higher the strength of market-incentivized environmental regulation.

#### Control variables

This article selected control variables at both the firm level and the city level to mitigate the potential influence of other factors. This has been reflected in previous studies [52, 53], including the following: The ratio of net profit to total assets is used to represent the return on assets of the company (*ROA*). A higher profitability level for the company is beneficial for green transformation and innovation. The ratio of management expenses to total operating income represents the agency costs (MC) of the company. The government intervention (*Gov*) is expressed as the ratio of local fiscal expenditures to the GDP of the province. The tangible asset ratio (*Tang*) is represented as the proportion of net fixed assets to the total assets of the firm. The age of a company (*age*) is represented by the number of years since its establishment [54].

As a result, the index system for all variables was obtained in Table 1. Table 2 provides the descriptive statistics of the variables.

**Table 1:** Variable definition.

Variable Type	Variable	Variable Symbols	Variable Meaning
Dependent variables	The level of transformation and innovation of enterprises	*TL*	R&D investment/total operating revenue
independent variable	Green Financial Support	*GreenFin*	The proportion of interest expenses of non-energy-consuming industries to total industrial interest expenses in the province (city, autonomous region) where the enterprise is located
Intermediary variables	Command-based environmental regulation	*ERc*	The "three wastes" index is constructed by the entropy value method
	Market Incentive-based Environmental Regulation	*ERm*	Sewage charges
Control variables	Return on Assets	*ROA*	Net profit/total assets
	Agency Costs	*MC*	Administrative expenses/total operating income
	Government intervention	*Gov*	Local fiscal expenditure / GDP of the province
	Tangible assets rate	*Tang*	Net fixed assets/total assets
	Year of business establishment	*age*	Number of years of business establishment

**Table 2:** Descriptive statistics.

Variable	N	Mean	Median	SD	Min	Max	VIF
*TL*	2310	3.369	3.422	2.395	0.010	12.240	
*GreenFin*	2310	0.524	0.512	0.140	0.207	0.808	1.040
*ROA*	2310	0.032	0.032	0.060	-0.266	0.175	1.060
*MC*	2310	0.075	0.058	0.067	0.007	0.441	1.130
*Gov*	2310	0.162	0.147	0.059	0.059	0.368	1.320
*Tang*	2310	0.265	0.263	0.143	0.011	0.646	1.070
*age*	2310	22.918	23.000	4.678	13.000	34.000	1.020
*ERc*	2310	0.835	0.671	0.704	0.000	2.585	1.740
*ERm*	2310	11.12	11.000	0.826	9.009	12.790	1.930

## 4. Results and discussions

### 4.1 Baseline regression and indirect effects test

Table 3 shows the study of the relationship between the support of green finance (*GreenFin*) and the level of transformation and innovation (*TL*) of enterprises. The first column does not contain control variables, and the coefficient of the effect of green finance support on the level of transformation and innovation of enterprises is 0.417 at the 10% level of significance. In regression (2), a control variable is introduced to improve the precision of the regression, and the regression coefficient is statistically significant at the 5% level. Overall, regardless of whether control variables exist, the support of green finance effectively promotes the transformation and innovation of high-energy-consuming enterprises, thus testing hypothesis H1.

**Table 3:** Baseline regression and test for mediating effects.

Variable	(1)	(2)	(3)	(4)	(5)	(6)
	*TL*	*TL*	*ERc*	*TL*	*ERm*	*TL*
*GreenFin*	0.417*	0.487**	0.078	0.474**	-0.541***	0.400*
	(1.7894)	(2.1141)	(1.6180)	(2.0598)	(-8.3514)	(1.7151)
*ER*				0.167**		-0.161*
				(2.0077)		(-1.9145)
*ROA*		-2.613***	-0.216	-2.577***	0.118	-2.594***
		(-3.8471)	(-1.3280)	(-3.8107)	(0.8198)	(-3.8284)
*MC*		6.584***	0.006	6.583***	0.131	6.605***
		(5.1734)	(0.0261)	(5.2000)	(0.5962)	(5.2041)
*Gov*		2.206**	-3.641***	2.815**	-0.631*	2.104**
		(2.1561)	(-9.5232)	(2.5553)	(-1.7547)	(2.0664)
*Tang*		0.163	0.053	0.154	0.026	0.167
		(0.3505)	(0.3811)	(0.3306)	(0.2110)	(0.3600)
*age*		0.258***	0.133***	0.236***	-0.029*	0.253***
		(7.8061)	(3.7926)	(6.8963)	(-1.7629)	(7.5862)
_cons	3.338***	-6.038***	-2.412***	-5.634***	12.478***	-4.023***
	(15.1993)	(-6.6808)	(-2.7199)	(-6.2060)	(24.2731)	(-2.8056)
Fixed effects	Year/lnd	Year/lnd	Year/lnd	Year/lnd	Year/lnd	Year/lnd
N	2310	2310	2310	2310	2310	2310
R^2^	0.839	0.851	0.844	0.851	0.897	0.851

Note: t statistics in parentheses

* p < 0.1

** p < 0.05

***p < 0.01.

From regressions (3), (4), (5), and (6), we can see that there is no mediating effect of the command-based environmental regulation, but slowing down the intensity of this environmental regulation can significantly promote the transformation and innovation of high-energy-consuming enterprises. Contrary to the findings of previous research [55], it suggests that green finance cannot promote green technology innovation through "market-driven" environmental regulation. Market-incentivized environmental regulations have an indirect effect by slowing down environmental regulations to slow down the cost of emissions and the cost of high energy consumption itself, so that they can carry out their own transformation and innovation to a greater extent. It may be that excessive taxation and penalties such as sewage charges exacerbate the financial burden and production costs of energy-intensive enterprises, which need to circumvent certain environmental regulations if they want to further increase their R&D investment to achieve transformational innovation. Green finance is based on traditional finance and is stimulated and led by the government. While giving financial support, it will reasonably optimize the corresponding regulation and further promote the transformation and innovation of high energy-consuming enterprises, partially testing hypothesis H2.

### 4.2 Endogeneity test

In this study, the instrumental variables approach was used to address possible endogeneity problems due to omitted variable bias, panel bias, etc. Although individual and time effects are controlled for in the above baseline regression, unobservable confounding factors may still be present. Based on this, drawing inspiration from previous research [56], the three surrounding provinces that are closest to the GDP size of province j where firm i is located are identified, and the mean value of green financial support in these three provinces in year t is used as the instrumental variable. Table 4 shows that the coefficient of green finance is still significant, which indicates that green finance can greatly contribute to the innovation transformation and upgrading of enterprises. This is in agreement with the results of the baseline regression and further validates hypothesis H1.

**Table 4:** Endogeneity regression results.

Variable	(1)	(2)
	*GreenFin*	*TL*
*GreenFin*		3.039***
		(0.592)
*greenfin*	0.763***	
	(0.024)	
Constant	0.173***	2.950***
	(0.020)	(0.449)
control variables	Y	Y
N	2,310	2,310
R^2^	0.316	0.191

Note: Standard errors are in parentheses.

* p < 0.1

** p < 0.05

***p < 0.01

### 4.3 Robustness tests

**Fig 2:**
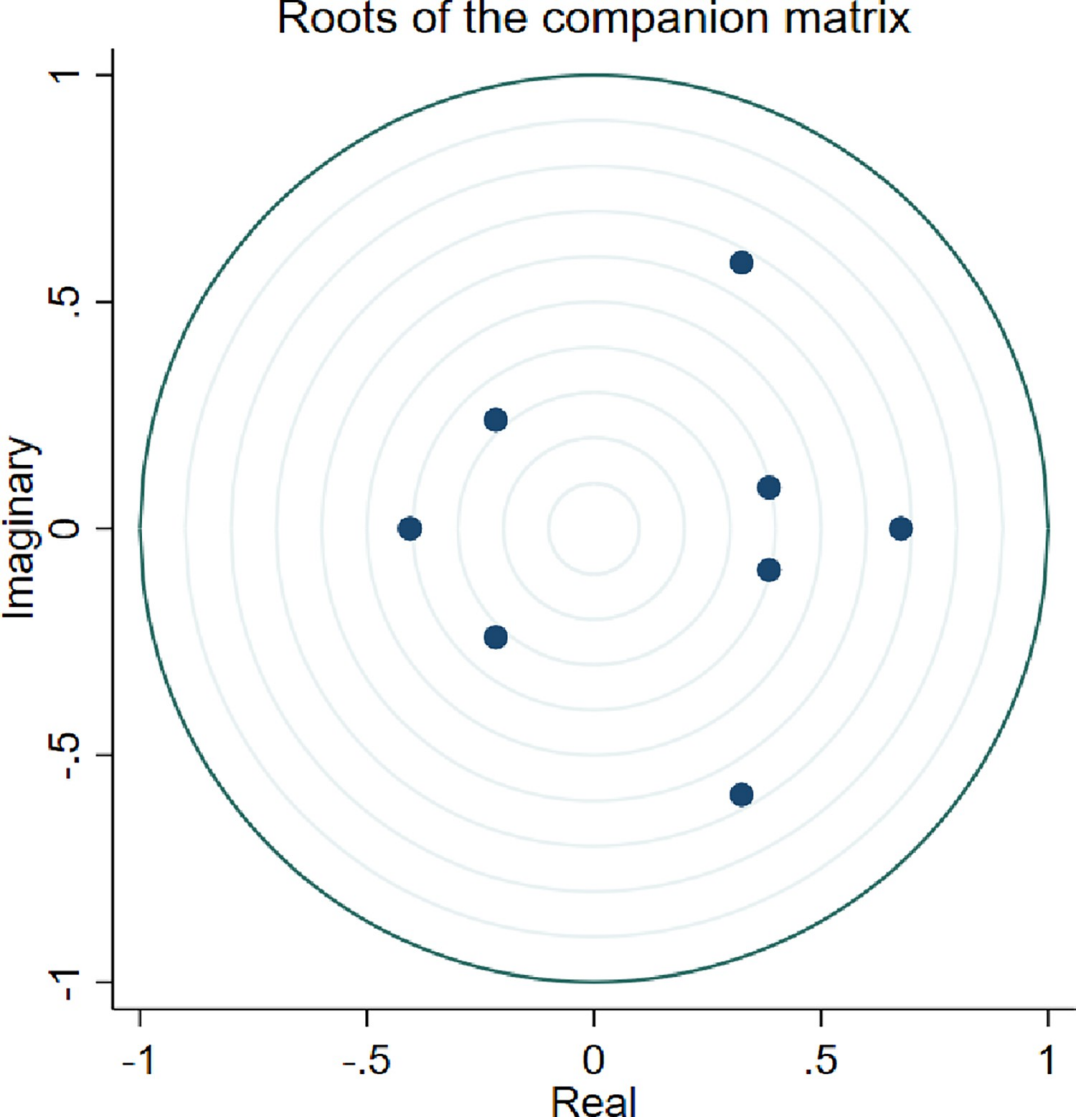
Schematic diagram of the roots of the accompanying matrix.

First, since the independent and dependent variables in this paper are at the provincial level and the firm level, respectively, to further exclude possible endogeneity effects triggered by reverse causality. The Granger Causality Test is now conducted, as shown in Fig 2 and Table 5. The results show that the characteristic roots are all within the unit circle, and the level of transformation and innovation of enterprises is not a Granger cause of green financial support, i.e., there is no reverse causality between them.

**Table 5:** Granger Causality Test results.

Equation	Excluded	Chi2	df	Prob>chi2
*TL*	GreenFin	5.7467	4	0.219
*TL*	ALL	5.7467	4	0.219

Second, shorten the time between samples. To avoid possible noise interference from the policy of the Industrial Green Development Plan introduced in 2016, the article shortens the sample period from 2017 to 2020. The results obtained are shown in (1) in Table 6, where the green financial support strength promotes the transformation and innovation of high energy-consuming enterprises at a significant level of 5% and the correlation coefficient is positive (0.958). This is in accordance with the preceding paper and indicates the robustness of the benchmark regression findings.

**Table 6:** Robustness test results.

Variable	(1)	(2)	(3)
	Reduced sample time	t+1 period	Adding control variables
*GreenFin*	0.958**	0.905**	0.488**
	(2.3589)	(1.9964)	(2.1017)
*ROA*	-2.316***	-0.814	-2.657***
	(-3.1264)	(-1.1235)	(-3.9306)
*MC*	6.443***	-1.160	7.089***
	(4.1603)	(-0.7743)	(5.5480)
*Gov*	1.873	-0.240	2.108**
	(1.3675)	(-0.1924)	(2.0453)
*Tang*	-0.021	1.041	0.090
	(-0.0374)	(1.5931)	(0.1958)
*age*	0.277***	0.300***	0.306***
	(7.6938)	(6.9700)	(6.2711)
*Size*			0.231*
			(1.8529)
*top*			0.003
			(0.4007)
_cons	-6.828***	-6.636***	-13.168***
	(-7.6548)	(-5.6102)	(-3.6965)
Fixed effects	Year/lnd	Year/lnd	Year/lnd
N	1848	1848	2310
R^2^	0.876	0.869	0.852

Note: t statistics in parentheses.

* p < 0.1

** p < 0.05

***p < 0.01.

Third, the percentage of R&D investment by enterprises using a lagging period Taking cues from the approach adopted by He and Tian [57], Column (2) of Table 6 shows that the relationship is still positive and significant, further solidifying the previous regression results.

Fourth, considering that larger companies tend to be more inclined to engage in innovative activities and face fewer external financial constraints [58], shareholders also play a crucial role within the organization. This study incorporates additional control variables for firm size (*Size*) and shareholder rights (*top*) to reduce potential endogeneity and unpredictability. The natural logarithm of the total assets of the firm and the shareholding of the top ten shareholders of the firm are used to represent these two control variables, respectively, and are added to the regression equation for regression. Column (3) of Table 6 shows that the result remains significantly at the 5% level, again confirming the prior conclusion and further validating the soundness of the prior regression findings.

### 4.4 Heterogeneity analysis

(1) Heterogeneity of enterprise size. The larger a company is, the more R&D investments it tends to be able to make, and the more innovative its transformation will be. In this paper, the median size of enterprises is used as the dividing line, and enterprises are divided into two groups: large enterprises and small and medium enterprises. In Table 7 (1) and (2) show that large enterprises are positive at the 1% significance level, while small and medium-sized enterprises fail to pass the test. It may lie in the fact that the economies of scale of large energy-consuming enterprises allow them to have a higher survival rate and easier access to external resources, providing more support for their own transformation and innovation.

**Table 7:** Heterogeneity test results.

Variable	Enterprise size		Corporate Ownership		Different low-carbon pilot areas	
	Large Companies	Non-Large Enterprises	Non-State Owned Enterprises	State-owned enterprises	Low carbon pilot area	Non-Low Carbon Pilot Areas
*GreenFin*	0.919***	0.072	0.641*	0.343	1.026*	0.347
	(3.385)	(0.176)	(1.810)	(1.118)	(1.917)	(1.461)
_cons	-6.844***	5.518***	-5.390***	6.027***	-18.822	-54.848***
	(-5.423)	(6.001)	(-5.512)	(4.148)	(-1.351)	(-7.044)
Control variables	Y	Y	Y	Y	Y	Y
Fixed effects	Year/lnd	Year/lnd	Year/lnd	Year/lnd	Year/lnd	Year/lnd
N	1046	1264	1426	884	516	1794
R^2^	0.878	0.842	0.843	0.839	0.845	0.854

Note: t statistics in parentheses.

* p < 0.1

** p < 0.05

***p < 0.01.

(2) Heterogeneity of business ownership. The level of support and social resources available to the company will vary depending on its ownership. Columns (3) and (4) in Table 7 show that green financial support has a greater influence on the transformation and innovation capacity of non-state enterprises, while it does not have a significant effect on state-owned enterprises. With the continued promotion of green finance and the introduction of relevant laws, non-state enterprises are receiving more attention and need to consider self-sufficiency. Non-state-owned enterprises generally exhibit weaker production capabilities compared to state-owned enterprises [59]. They have to adapt to the needs of society and control the cost of environmental protection, so they require added investment in research and development and efficient use of resources to transform and innovate, so the need to achieve transformation and innovation is more obvious.

(3) Heterogeneity of low-carbon pilot regions (In 2010, the National Development and Reform Commission issued the Notice on the Piloting of Low-Carbon Provinces and Low-Carbon Cities, which identified the five provinces of Guangdong, Liaoning, Hubei, Shaanxi, and Yunnan and the eight cities of Tianjin, Chongqing, Shenzhen, Xiamen, Hangzhou, Nanchang, Guiyang, and Baoding as the first low-carbon pilot cities in China, and added 28 cities in one province and 45 cities in 2012 and 2017 respectively.). In this paper, the five provinces that were first identified as low-carbon pilot regions, as well as Tianjin and Chongqing, are selected as low-carbon pilot regions, while the rest are non-low-carbon pilot regions. The results reveal that the intensity of green financial support has a solid impact on the level of corporate transformation and innovation (the correlation coefficient is 1.026), and it’s statistically significant. It may be that the low-carbon pilot regions advocate green development, which directly causes a dramatic escalation in the cost of survival for energy-intensive enterprises. To adapt to the green development policy, high-energy-consuming enterprises have to invest more in R&D and show a more urgent willingness to transform and innovate, thus significantly improving their own transformation and innovation capabilities.

### 4.5 Mechanism analysis

Previous research indicates that the internal financing of companies often falls short of meeting the financial needs for green research and development, while external financing constraints significantly limit firms’ research and development activities [60]. The first column of Table 8 reveals that the regression coefficient of the interaction term is -1.732. It shows that the greater the financing constraint of high energy-consuming enterprises, the more likely it is that green finance will curtail the contribution of green finance to the transformation and innovation of high energy-consuming enterprises. The development of green finance provides diversified financing channels for enterprises, thus alleviating their financing constraints, giving them more funds for R&D activities, and improving their ability to transform and innovate.

**Table 8:** Mechanism analysis results.

Variable	(1)	(2)
	*TL*	*TL*
*FC*	0.643	
	(1.523)	
FC*GreenFin	-1.732**	
	(-2.517)	
*GS*		-0.093**
		(-2.030)
GS*GreenFin		0.171**
		(2.019)
_cons	-6.306**	-4.650***
	(-6.805)	(-4.093)
Control variables	Y	Y
Fixed effects	Year/lnd	Year/lnd
N	2310	2300
R^2^	0.852	0.852

Note: t statistics in parentheses

* p < 0.1

** p < 0.05

***p < 0.01.

Government subsidies facilitate energy-intensive enterprises to mitigate the risk of externalities in the process of transforming and innovating, thus driving them to engage in intensive innovation activities. Column (2) shows the interaction term significantly affects the level of transformation and innovation of high energy-consuming enterprises at the 5% level (the correlation coefficient is 0.171), and the effect is positive. It may be because the government gives the enterprise a generous subsidy to make the enterprise have a large number of cash holdings, thus helping the enterprise squeeze out the existing funds and thus increasing the enterprise’s transformation and innovation upgrading efforts. The previous hypothesis H3 is verified.

## 5. Discussion

### 5.1 Benefits analysis of green financial development for transformation and innovation of high energy-consuming enterprises

As an important force in mitigating environmental pollution, green finance plays an influential role in facilitating environmental protection and reducing environmental pollution. Meanwhile, he also enhances the willingness of energy-consuming enterprises to transform and innovate, thus improving their financial performance and achieving "win-win" economic and environmental benefits. To thoroughly study the economic and environmental benefits of green finance on the transformation and innovation of high energy-consuming enterprises, the following economic model was established:

$${TBQ}_{it}=\alpha +\delta_{1}{GreenFin}_{it}+\delta_{2}{Control}_{it}+\varphi_{i}+\gamma_{t}+\varepsilon_{it}$$ (6)

$${EPI}_{it}=\alpha +\theta_{1}{GreenFin}_{it}+\theta_{2}{Control}_{it}+\varphi_{i}+\gamma_{t}+\varepsilon_{it}$$ (7)

In this model, TBQ indicates the financial performance of high energy-consuming enterprises, measured by Tobin’s Q; EPI indicates the environmental performance of high energy-consuming enterprises, which is selected as the ratio of the enterprise’s emission fee to operating income in the current year; and the remaining variables are defined regarding Table 1.

Table 9 shows that the coefficient of *GreenFin* remains significant (the correlation coefficient is 0.347), which indicates that green finance can lead to an improvement in the value of the company and its financial performance. However, in terms of environmental benefits, the advancement of green finance does not show a significant correlation with the environmental benefits resulting from enterprises’ transformative innovation activities. This facilitation may be mainly due to the fact that green finance has prompted companies to innovate in green transformation, answering the call of the current times and thus contributing to the ability of companies to gain the trust of financial institutions more easily. Colaco and Simão [61] focused on the economic behavior of enterprises and found that green finance encourages companies to provide greater economic support for the environment and society, thus enhancing environmental performance. Additionally, incentivizing green production within companies leads to increased green financing, which positively contributes to environmental performance [62]. However, the absence of notable environmental benefits in this study may be attributed to the incomplete development of green finance in China at its current stage. And it is still difficult for enterprises to achieve real transformation and innovation, which leads to the fact that enhancing the environmental efficiency of enterprises is not significant at this stage. Similar findings have been obtained by previous scholars [63], which verifies the previous hypothesis H4.

**Table 9:** The economic and environmental benefits of green finance for transformative innovation.

Variable	(1)	(2)
	*Tobin′s Q value*	*EPI*
*GreenFin*	0.347*	0.043
	(1.6791)	(1.4842)
_cons	1.629**	-0.120**
	(2.0490)	(-2.1132)
Control variables	Y	Y
Fixed effects	Year/lnd	Year/lnd
N	2095	2310
R^2^	0.707	0.659

Note: t statistics in parentheses.

* p < 0.1

** p < 0.05

***p < 0.01.

### 5.2 Comparing with existing studies

To ensure the accuracy and reliability of the results obtained in this study, we conducted a thorough comparison with previous research. The advancement of green finance has been shown to be beneficial for the green transformation and innovation of enterprises, as evidenced by various studies [64–66], Wang et al. [37] have observed that in developing countries with lower levels of financial development, green finance facilitates the initiation of green innovation activities. However, it proves detrimental to countries with already well-established environmental development in the pursuit of green innovation. In contrast to the findings of this study, existing research indicates that stringent regulatory oversight, when excessively coercive in nature, can hinder the development of green finance. This phenomenon also represents a significant impediment to the advancement of green innovation in Asian countries [51]. Zhang [67] found that green credit policies promote environmentally-induced research and development, which may increase innovation inputs and hinder transformative innovation for companies. The discrepancy in these findings may be attributed to green finance’s impact on constraining funds and compressing companies’ loan stocks [68], as well as limiting investment and causing insufficient liquidity for transformative innovation in companies, which also brings significant uncertainty to their innovation [69]. Under strict environmental regulations, companies tend to favor low-risk green projects [70], which also encourages them to fully utilize green finance. This study also found that alleviating financing constraints for high-energy-consuming enterprises is beneficial for the transformative innovation role of green finance. In previous research, Liu et al. [36] similarly found that green loans can effectively reduce financing constraints for companies in energy-intensive industries. Additionally, government funding subsidies facilitate the implementation of a series of green innovation decisions by companies, thereby promoting transformative innovation outcomes [71], which aligns with the findings of this study.

## 6. Conclusions and policy recommendations

This paper explores the impact of the development of green finance on the transformation and innovation of high energy-consuming enterprises based on data on the transformation and innovation of 462 high energy-consuming enterprises during 2016–2020 and data on the level of green financial support in each province. The results are as follows: Firstly, Overall, the strong support of green finance significantly promotes the transformation and innovation upgrading of high energy-consuming enterprises, and market-incentivized environmental regulation plays a partial mediating role between green finance and the transformation and innovation of high energy-consuming enterprises. Additionally, the results of the heterogeneity analysis indicate that large, high-energy-consuming enterprises, non-state-owned enterprises, and high-energy-consuming enterprises in low-carbon pilot regions are more significantly affected. Lastly, the mechanism test finds that the development of green finance can use government subsidies and mitigate financing constraints to promote the transformation and innovation of high-energy-consuming enterprises. In addition, the development of green finance can significantly improve the financial performance of high-energy-consuming enterprises. The scope of this study is confined to the impact of green finance on high-energy-consuming enterprises. However, its applicability to other industries or businesses remains limited, posing certain research constraints. Additionally, due to measurement errors in the data and limitations in research methods, it is possible that this study may underestimate the net benefits of green finance on the transformation and innovation of high-energy-consuming enterprises.

Based on the above conclusions, this paper makes the following recommendations: Firstly, it is crucial to increase support for green finance policies by allocating more financial resources and capital to industries with lower energy consumption and less pollution. simultaneously enhancing the efficiency of green finance resources and actively guiding high-energy-consuming enterprises towards green transformation and innovation while providing appropriate financial support to facilitate their efforts. Secondly, raising the financing threshold for high energy-consuming enterprises to prompt more high energy-consuming enterprises to transform and innovate in the direction of green production and green operation and also strengthening the real-time inspection of the operation and environmental protection activities of high energy-consuming enterprises will help develop a more reasonable environmental regulation intensity that fully takes into account the environmental protection costs of high energy-consuming enterprises. Thirdly, to create a stable external environment for green finance to support the transformation and upgrading of high energy-consuming enterprises, the government should strengthen the development of low-carbon pilot regions, appropriately abolish credit restrictions on non-state enterprises, and encourage them to continue to increase investment in R&D. When implementing green finance policies, dynamically adjust the intensity of relevant incentives and penalties for high-energy-consuming enterprises of different regions, sizes, and ownership systems.

The limitations of this study and future research directions are as follows: Firstly, this study only explores the impact of green finance on the transformative innovation of high-energy-consuming enterprises and does not examine its effects on other aspects of these enterprises. Further investigation is needed to understand the influence of green finance on the performance and corporate social responsibility of high-energy-consuming enterprises. Secondly, the transmission pathways through which green finance affects high-energy-consuming enterprises require further research (e.g., internal organizational structure, green acquisitions). The relationship between green credit, green investments, and high-energy-consuming enterprises, as important components of the green finance system, also warrants exploration. Lastly, due to data availability limitations, the scope of our study is limited. Future research should apply the research framework and approach to a broader range of countries and high-energy-consuming enterprises to enhance the scientific value of this study.
